# Race and region have independent and synergistic effects on dietary intakes in black and white women

**DOI:** 10.1186/1475-2891-11-25

**Published:** 2012-04-13

**Authors:** P K Newby, Sabrina E Noel, Rachael Grant, Suzanne Judd, James M Shikany, Jamy Ard

**Affiliations:** 1 Department of Pediatrics, Boston University School of Medicine, Boston, MA 02118, USA; 2 Department of Epidemiology, Boston University School of Public Health, Boston, MA 02118, USA; 3 Program in Graduate Medical Nutrition Sciences, Boston University School of Medicine, Boston, MA 02118 USA; 4 Program in Gastronomy, Culinary Arts, and Wine Studies, Boston University Metropolitan College, Boston, MA 02215, USA; 5 Department of Biostatistics, School of Public Health, University of Alabama at Birmingham, Birmingham, AL 35294-0022, USA; 6 Division of Preventive Medicine, School of Medicine, University of Alabama at Birmingham, Birmingham, AL 35205, USA; 7 Department of Nutritional Sciences, School of Medicine, University of Alabama at Birmingham, Birmingham, AL 35294-3412, USA

**Keywords:** Diet, Health disparities, Nutrients, Blacks, Race, Region

## Abstract

**Background:**

Few studies have examined the effects of race and region on dietary intakes and the evidence on racial and regional disparities among women is limited. We aimed to examine whether race and region were associated with nutrient intakes among black and white women living in the Stroke Belt, Stroke Buckle, and Other regions in the United States. We hypothesized that significant differences would be observed among population sub-groups and that the effects of race on dietary intakes would vary across regions.

**Methods:**

This study included dietary data from 12,105 women from the Reasons for Geographic and Racial Differences in Stroke study (United States). Dietary data were collected using the Block 98 food frequency questionnaire.

**Results:**

Blacks consumed 1.05% lower energy from saturated fat (95% CI: -0.95, -1.16), and intakes were also lower in the Buckle (β = -0.20; 95% CI: -0.08, -0.32) and Belt (β = -0.35; 95% CI: -0.24, -0.46) compared to the Other regions. Within each region, sodium, potassium, and magnesium intakes were all lower among black women compared to white women (*P *<0.05 for all); intakes were significantly lower among blacks living in the Belt and Buckle compared to those in the Other regions. Significant interactions between race and region were detected for trans fat, calcium, and cholesterol (*P *<0.05 for all), where black women in the Other regions consumed the lowest dietary cholesterol and calcium while black women in the Belt consumed the lowest trans fat.

**Conclusions:**

Race and region were significantly associated with nutrient intakes in a large study of black and non-Hispanic white women in the United States. Intakes of trans fat, calcium, and cholesterol among black and white women differed across regions. Race and region thus interact to impact dietary intakes, and their effects may be mediated by such factors as the broader food environment and food availability as well as food customs and culture. Race, region, and their correlates should therefore be considered together when examining diet and disease associations and planning dietary advice for population sub-groups.

## Background

In the United States, a higher prevalence of chronic diseases such as hypertension, stroke, and diabetes has been observed in Southern populations, particularly among blacks [1,2]. In addition, a variety of dietary disparities between non-Hispanic whites and blacks has been detected, including higher intakes of dietary cholesterol and lower intakes of potassium, fiber, fruits, and vegetables in blacks [3,4]. Regional differences in diet also have been seen in the United States. For example, individuals in the South and West consume more dietary cholesterol than those in the North or the East, and Southerners consume the lowest amount of fiber compared to other regions [5].

Several studies have examined dietary intakes across either racial/ethnic or regional groups in the United States. In an early investigation, black women aged 25-75 y consumed less saturated and total fat than whites in the National Health and Nutrition Examination Survey (NHANES) II (1976-1980) [6]. More recently, a small study of black and white middle-aged women in Louisiana showed that black women consumed less fiber, protein, calcium, magnesium, linoleic acid, arachidonic acid, and eicosapentanoic acid while total energy, fat, and carbohydrate intakes were similar [7]. In the Mississippi Delta, black adults consumed a less optimal diet than whites although all had poor adherence to dietary recommendations [8]. In NHANES III (1988-1994), Southerners consumed more monounsaturated fat, sodium, and cholesterol and less fiber, potassium, calcium, and magnesium compared to other regions in the United States [5]. However, few differences between black and white urban Southern women were observed in another study [9]. While differences in findings may reflect a cohort effect and other differences in study design and samples, additional work is needed to elucidate the associations and interactions between race, region, and diet, which have important implications for identifying dietary risk factors that may contribute to health disparities.

Our objective was to describe dietary intakes of women currently enrolled in the Reasons for Geographic and Racial Differences in Stroke (REGARDS) study and determine whether there were race and regional differences in macro- and micronutrient intakes among black and white women living in the Stroke Belt (non-coastal regions of North Carolina, South Carolina, and Georgia, as well as Alabama, Arkansas, Georgia, Louisiana, Mississippi, and Tennessee) and Stroke Buckle (coastal plain regions of North Carolina, South Carolina, and Georgia) compared to those living in the Other regions of the United States. An additional goal was to examine whether race and region were independently related to selected nutrient intakes associated with health promotion and disease prevention and whether the two would interact synergistically. We hypothesized that significant differences would be observed among population sub-groups and that the effects of race on dietary intakes would vary across regions.

## Methods

### Study population and sample

Participants came from REGARDS, a national longitudinal cohort study which enrolled approximately 30,000 black (≈41%) and white (≈59%) individuals aged 45 years and older between 2003 and 2007; this paper examines dietary intakes at baseline. The study oversampled individuals in the Stroke Belt (20%) and Stroke Buckle (30%), and 50% came from the other 48 contiguous United States. Within each region, individuals were recruited via mail and telephone using commercially available lists of residents. After an initial telephone interview to obtain baseline demographic, socioeconomic, and health information, an in-home examination was conducted by trained personnel. At that appointment, the Block 98 food frequency questionnaire (FFQ) was left with participants to be self-administered and returned to the coordinating center. Informed consent was obtained from all participants, and all methods for the parent study were reviewed and approved by the Institutional Review Board for Human Use at the University of Alabama at Birmingham. The IRB at Boston Medical Center/Boston University School of Medicine also approved the protocol for this ancillary study.

This study included only women who returned a completed FFQ (n = 13,433), which was approximately 81% of the total sample of women in the main study. Of these, we excluded individuals who left more than 15% of food items blank (n = 965) and those with implausible values for energy intake (<2093 kJ or >18841 kJ, n = 357). After all exclusions, data from 12,105 women were available for the analysis.

### Exposure and covariate assessment

Demographic information and medical history were obtained via computer-assisted telephone interview (CATI). Average annual dietary intakes were collected via self-administered questionnaire, the Block 98 FFQ. Details on how to complete dietary questionnaires were provided to participants, and questionnaires were mailed back to the study center at the University of Alabama at Birmingham.

Race and region were our primary exposure variables. Race was determined via self-report on the CATI, where participants self-identified themselves as either black or white; a category for mixed race was not provided. Individuals who self-identified themselves as Hispanic during follow-up interviews were excluded from the study. Regions were defined as the Stroke Buckle ("Buckle" = coastal plain region of NC, SC and GA), the Stroke Belt ("Belt" = remainder of NC, SC, and GA, plus AL, MS, TN, AR, and LA), and Other ("Other" = remaining states from the contiguous United States).

Sociodemographic covariates were assessed from both the CATI and mail-in FFQ and categorized in this study as follows: age (y), sex (male/female), income (<$25,000, $25,000 to ≤ $50,000, >$50,000 to ≤ $75,000, and > $75,000), education (<high school, high school graduate, some college, college degree or higher), marital status (single, married, divorced, widow, other), smoking status (current, never, former), multivitamin supplement use (% yes), hormone therapy use (% yes), television watching (0 h/wk, 1-6 h/wk, 1 h/d, 2-3 h/d, >3 h/d), and physical activity (0 times/wk, 1-3 times/wk, ≥4 times/wk). Physical activity was assessed through questionnaire as the self-reported number of times per week an individual participated in intense activity.

Anthropometric and physical measurements, including height, weight, waist circumference, and blood pressure, were performed by trained technicians at an in-home examination. Height was measured using an 8' metal tape measure and square and weight was measured using a calibrated, digital scale (Salter, Salter Brecknell, Fairmont, MN). Waist circumference was assessed using a cloth tape measure at the midpoint between the lowest rib on the right side and the top of the iliac crest by trained technicians. Blood pressure measures were performed in duplicate using an aneroid sphygmomanometer. BMI was calculated from measured weight and height (kg/m^2^). Presence of medical conditions was determined following standard assays, as described elsewhere [10], and included diabetes (fasting plasma glucose >6.99 mmol/L, non-fasting glucose ≥11.1 mmol/L, or use of diabetes medication), hypertension (systolic blood pressure ≥140 mmHg, diastolic blood pressure ≥90 mmHg, or use of antihypertensive medications), and hypercholesterolemia (total cholesterol ≥6.216 mmol/L, LDL cholesterol ≥4.144 mmol/L, HDL cholesterol ≤1.036 mmol/L, or use of lipid-lowering medications). Additional methodological details of the REGARDS study are provided elsewhere [10].

### Dietary assessment

Dietary intakes were assessed using the self-administered, semi-quantitative Block 98 FFQ [11] designed to measure usual dietary intakes. For each item on the FFQ, a common serving size of the food or beverage is specified (e.g., ½ cup carrots), and participants are asked how often they consumed this amount on average during the previous year. Individuals selected from 9 possible frequencies ranging from "never or less than once per month" to "1 (or 2) or more times per day" and selected the appropriate portion size. Portion size for unitary items was queried ordinally as "1, 2 or 3" and the number consumed each time was reported. For non-unitary foods, a photo was provided to aid in estimating four different portions. For each food, an amount was assigned based on the gram weight of the volume for the selected portion size model.

FFQs were completed by participants at home and mailed to the study center, where they were checked for completeness and scanned. Scanned FFQ files were sent to NutritionQuest™ (Berkeley, CA) for processing. Nutrients were calculated using the Block nutrient database, which was developed from the USDA Nutrient Database for Standard Reference [12] and other sources (e.g., manufacturers' data); values for trans fat [13,14] and other nutrients were identified from available sources in the published literature and through manufacturer's data. The amount of each food consumed was calculated by multiplying the reported frequency by the portion size for each food item. The total amount of a contributing nutrient from each food was derived by multiplying the amount consumed by the amount of the nutrient in the given FFQ line item (from the Block nutrient database). Nutrients were summed over all FFQ food items to provide estimates for total daily nutrient intakes; intakes from dietary supplements were not included (personal communication, Torin Block, NutritionQuest™, Berkeley, CA). Although the Block 98 FFQ has not yet been validated in the REGARDS population, deattenuated Pearson correlation coefficients were moderate to high (median = 0.59) in a validation study in Canadian women [15].

### Statistical analyses

For all descriptive analyses, we stratified our sample by region and by race within region *a priori*, as our study objective was to examine differences across these population sub-groups. We calculated sample means and standard deviations and frequencies for sociodemographic, anthropometric, and nutrient variables. Chi square analyses were used for categorical variables and analysis of variance (ANOVA) for continuous variables to test for differences in racial sub-groups within regions. Nutrient intakes were adjusted for total energy using the nutrient residual approach [16]; macronutrients were treated as percent energy. As dietary intakes were not normally distributed, medians and inter-quartile ranges were calculated for nutrients and significant differences between races within region were tested using the Wilcoxon two sample *t*-test. Significant differences across the three regions were tested using the Kruskal-Wallis test.

Descriptive analyses of dietary intakes included energy, macronutrient, and micronutrient intakes. We then performed linear regression analyses to examine whether race and region were associated with selected nutrients that have been associated with health promotion and disease prevention, as follows: fiber (g), saturated fat (percent energy), trans fat (percent energy), sodium (mg), potassium (mg), magnesium (mg), calcium (mg), and dietary cholesterol (mg). Although nutrient intakes were not normally distributed, intakes were not transformed as this violation of normality did not appear to compromise model fit and facilitates comparison with our companion paper in men [17]. For each nutrient outcome, we created two multivariable adjusted models. The first model was adjusted for age, total energy, BMI, multivitamin use, menopausal status, hormone therapy use, income, education, marital status, smoking status, alcohol use, physical activity, television viewing, hypertension, dyslipidemia, and diabetes. The proportion of missing data in this study was low (<2% for all covariates with the exception of income, which was <14%), and dummy variables were used to include those with missing covariate information. The second model tested for effect modification by adding an interaction term for race and region (race*region) to the multivariable-adjusted model. We performed stratified analyses by race and region for any interaction terms with *p *values <0.05.

Alpha was set at *p *< 0.05 for all analyses and all hypothesis tests were two tailed. Statistical analyses were performed using the Statistical Analysis System (SAS) for Windows, Version 9.2 (SAS Institute Cary, NC) [18].

## Results

In the Buckle and Belt, black women were younger than white women (Buckle: 62.2 ± 8.9 y vs. 64.4 ± 9.4 y; Belt: 62.4 ± 8.7 y *vs*. 64.5 ± 9.2 y; *p *< 0.001 for both); there was no significant difference in age across racial groups in the Other regions (*p *= 0.06) (Table 1). Within each region, a higher proportion of white women were post-menopausal, used hormone therapy, and consumed multivitamin supplements compared to black women (*p *< 0.05 for all). A significantly smaller proportion of black women within each region had an annual income ≥ $75,000/year and a college degree or higher (*p *< 0.001 for all). Within each region, a higher percentage of blacks viewed television >3 h/d compared to white women (≈42% vs. ≈25%, respectively). With the exception of physical activity (*p *= 0.22), significant differences between regions were observed across all demographic and lifestyle characteristics.

**Table 1:** Demographic and lifestyle characteristics of 12,105 women participating in the Reasons for Geographic and Racial Differences in Stroke study, stratified by race and geographic region^1^

**Demographic and lifestyle characteristics**	**Stroke Buckle**			**Stroke Belt**			**Other** **(non-stroke buckle or belt)**			***P *value between regions**
	**Black** **(n = 971)**	**White** **(n = 1,905)**	***P*** **value**	**Black** **(n = 1,600)**	**White** **(n = 2,603)**	***P*** **value**	**Black** **(n = 2,237)**	**White** **(n = 2,789)**	***P*** **value**	
**Age, y, mean ± SD**	62.2 ± 8.9	64.4 ± 9.4	<0.001	62.4 ± 8.7	64.5 ± 9.2	<0.001	64.5 ± 9.0	65.0 ± 9.6	0.06	<0.001
**Post-menopausal, %**	85.0	88.8	0.003	85.7	89.3	<0.001	88.4	90.2	0.03	0.04
**Hormone use, %**	46.5	68.8	<0.001	52.1	69.6	<0.001	45.8	60.5	<0.001	<0.001
**Multivitamin use, %**	40.7	51.1	<0.001	43.3	52.9	<0.001	43.3	57.2	<0.001	0.01
**Income, %**			<0.001			<0.001			<0.001	<0.001
**<$25,000**	40.9	23.6		45.3	26.5		37.3	23.3		
**$25,000-$50,000**	30.4	30.3		27.5	31.5		30.3	29.5		
**$50,000-$75,000**	9.0	14.0		9.7	12.5		11.7	14.2		
**≥$75,000**	5.7	17.1		5.2	14.4		8.4	17.7		
**Education, %**			<0.001			<0.001			<0.001	<0.001
**< High school**	19.3	7.6		19.6	8.4		12.1	4.8		
**High school graduate**	29.2	29.5		27.4	29.9		27.4	25.2		
**Some college**	25.3	28.7		25.8	29.6		32.5	29.5		
**College degree or higher**	26.1	34.2		27.3	32.1		28.1	40.6		
**Marital status, %**			<0.001			<0.001			<0.001	<0.001
**Single**	8.0	2.2		8.8	3.0		10.1	5.9		
**Married**	39.7	60.2		38.7	57.0		28.0	50.6		
**Divorced**	19.5	13.3		21.4	14.7		28.2	17.8		
**Widow**	27.1	23.3		27.4	24.5		29.5	24.8		
**Other**	5.7	0.9		3.7	0.8		4.2	0.9		
**Smoking, %**			<0.001			0.005			<0.001	<0.001
**Current**	11.0	13.3		17.0	13.4		17.5	12.1		
**Never**	61.2	53.3		53.3	54.9		45.8	53.4		
**Former**	27.9	33.4		29.6	31.7		36.7	34.5		
**Physical activity, %**			0.22			0.04			<0.001	0.24
**0 times/wk**	37.8	36.7		41.2	37.6		40.3	34.3		
**1-3 times/wk**	38.1	36.2		36.0	36.8		36.4	37.2		
**4 or more times/wk**	24.2	27.2		22.8	25.7		23.3	28.5		
**Television viewing, %**			<0.001			<0.001			<0.001	0.04
**0 h/wk**	0.2	0.8		0.6	1.0		0.6	1.1		
**1-6 h/wk**	13.5	13.6		14.0	13.6		12.1	14.8		
**1 h/d**	3.3	7.3		3.1	7.5		2.6	8.2		
**2-3 h/d**	41.0	53.5		36.1	51.7		38.7	51.1		
**>3 h/d**	42.1	24.8		46.2	26.4		46.1	24.8		

^1^Differences within (black *vs*. white) and between regions (Stroke Buckle *vs*. Stroke Belt *vs*. Other) were examined using t tests for continuous variables (age) and chi-square for categorical variables

Compared to white women, black women within each region weighed significantly more and had a larger waist circumference; differences were not statistically significant between regions for either variable (Table 2). A significantly larger proportion of black women were obese compared to white women; the highest prevalence of obesity was seen in the Buckle (58.5%), followed by the Belt (56.1%) and Other regions (52.8%). Blacks also had a higher prevalence of hypertension and diabetes within each region, although differences across region were only significant for diabetes (*p *< 0.001).

**Table 2:** Anthropometric characteristics and health indicators of 12,105 women participating in the Reasons for Geographic and Racial Differences in Stroke study, stratified by race and geographic region^1^

**Anthropometric characteristics and health indicators**	**Stroke Belt**			**Stroke Belt**			**Other** **(non-stroke buckle or belt)**			***P *value between regions**^2^
	**Black** **(n = 971)**	**White** **(n = 1,905)**	***P*** **value**	**Black** **(n = 1,600)**	**White** **(n = 2,603)**	***P*** **value**	**Black** **(n = 2,237)**	**White** **(n = 2,789)**	***P*** **value**	
**Weight, kg, mean ± SD**	87.6 ± 21	74.8 ± 17	<0.001	86.5 ± 21	74.3 ± 17	<0.001	84.3 ± 20	74.9 ± 18	0.001	0.92
**Height, cm, mean ± SD**	163.9 ± 7	163.1 ± 7	0.003	164 ± 7	163.2 ± 7	<0.001	163.1 ± 7	162.6 ± 7	0.02	<0.001
**Waist, cm, mean ± SD**	97.2 ± 15.8	88.4 ± 15.2	0.001	97.4 ± 15.7	88.1 ± 15.2	<0.001	96.1 ± 15.7	89.1 ± 16.2	<0.001	0.05
**BMI, kg/m^2^, mean ± SD**	32.5 ± 7.3	28.1 ± 6.2	0.001	32.1 ± 7.1	27.8 ± 6.2	<0.001	31.5 ± 7.1	28.2 ± 6.2	<0.001	0.19
**BMI weight group**			<0.001			<0.001			<0.001	
**Overweight, %**	27.7	32.4		30.2	32.9		32.0	34.7		0.02
**Obese, %**	58.5	32.7		56.1	30.1		52.8	31.7		
**Hypertension, %**	71.7	50.9	<0.001	74.5	48.2	<0.001	71.4	45.7	<0.001	0.58
**Diabetes, %**	30.9	13.8	<0.001	30.0	13.0	<0.001	24.8	9.7	<0.001	<0.001
**Dyslipidemia, %**	51.0	55.6	0.02	53.2	52.5	0.67	52.3	50.9	0.34	0.10

^1^Differences within (black *vs*. white) and between regions (Stroke Buckle *vs*. Stroke Belt *vs*. Other) were examined using t tests for continuous variables (age) and chi-square for categorical variables.

Compared to white women, black women within each region consumed higher amounts of carbohydrate and lower amounts of total fat, fiber, and alcohol (*p *≤ 0.005 for all) (Table 3). Regional differences in macronutrient and energy intakes were significant with the exception of percent of energy from total fat (*p >*0.05). Black women consumed more vitamin C than white women in all regions (*p *≤ 0.0002) while white women in the Buckle and Belt consumed less vitamin C compared to those in the Other regions (Buckle: 79.1 mg/d, Belt: 80.5 mg/d; Other: 93.2 mg/d; *p *< 0.0001 across regions) (Table 4). Black women consumed significantly lower intakes of calcium, magnesium, potassium, iron, and sodium within each region (*p *< 0.05 for all). Compared with white women, black women consumed more cholesterol in the Belt region (167 mg/d *vs*.156 mg/d, *p *= 0.002) and less cholesterol in the Other regions (152 mg/d *vs*.162 mg/d, *p *= 0.005). Black women in the Buckle region also had higher cholesterol intakes compared to white women (161 mg/d *vs*. 155 mg/d, *p *= 0.13).

**Table 3:** Daily energy and macronutrient intakes among 12,105 women participating in the Reasons for Geographic and Racial Differences in Stroke study, stratified by race and geographic region

**Energy and macronutrients (intakes/d)^1^**	**Stroke Buckle**			**Stroke Belt**			**Other** **(non-stroke buckle or belt)**			***P *value between regions^3^**
	**Black** **(n = 971)**	**White** **(n = 1,905)**	***P*** **value^2^**	**Black** **(n = 1,600)**	**White** **(n = 2,603)**	***P*** **value^2^**	**Black** **(n = 2,237)**	**White** **(n = 2,789)**	***P*** **value^2^**	
**Energy, kJ**	5813 (4000)	6039 (3222)	0.21	6154 (4016)	6266 (3418)	0.62	5920 (3739)	6203 (3001)	0.001	0.002
**Carbohydrate, g**	177 (119)	171 (95.9)	0.005	188 (120)	178 (100)	0.0001	178 (114)	173 (93.5)	0.002	<0.0001
**Carbohydrate, % energy**	51.6 (12.3)	48.1 (11.6)	<0.0001	50.9 (11.9)	48.5 (11.7)	<0.0001	50.7 (12.3)	47.1 (12.1)	<0.0001	0.001
**Protein, g**	47.8 (35.9)	51.9 (30.4)	0.0002	50.1 (35.5)	53.7 (33.3)	0.0003	49.0 (33.7)	56.1 (32.5)	<0.0001	0.0009
**Protein, % energy**	13.8 (4.1)	14.5 (3.8)	<0.0001	13.7 (4.0)	14.5 (4.1)	<0.0001	13.8 (4.0)	15.2 (4.0)	<0.0001	<0.0001
**Total fat, g**	54.6 (44.5)	59.6 (38.0)	0.0007	57.6 (45.0)	62.0 (41.3)	0.001	56.1 (42.5)	61.8 (38.9)	<0.0001	0.02
**Total fat, % energy**	36.0 (9.5)	37.9 (9.8)	<0.0001	36.2 (10.1)	37.8 (10.0)	<0.0001	36.6 (10.5)	37.9 (10.5)	<0.0001	0.81
**Saturated**	10.1 (3.0)	10.8 (3.4)	<0.0001	10.1 (3.0)	10.6 (3.3)	<0.0001	10.2 (3.2)	10.9 (3.8)	<0.0001	0.001
**Trans**	2.8 (1.5)	2.8 (1.5)	0.77	2.9 (1.5)	2.9 (1.6)	0.02	2.7 (1.5)	2.6 (1.4)	0.0006	<0.0001
**Monounsaturated**	13.3 (4.3)	14.0 (4.1)	<0.0001	13.3 (4.1)	14.0 (4.3)	<0.0001	13.5 (4.3)	14.2 (4.4)	<0.0001	0.03
**Polyunsaturated**	9.2 (3.7)	9.6 (3.8)	0.0003	9.5 (3.9)	9.6 (4.0)	0.007	9.4 (3.9)	9.2 (3.7)	0.002	<0.0001
**Fiber, g**	12.8 (9.6)	13.7 (9.2)	0.002	12.8 (9.5)	14.3 (10)	<0.0001	13.2 (9.9)	14.6 (9.5)	<0.0001	0.0001
**Alcohol, % energy**	0.0 (0.4)	0.2 (1.7)	<0.0001	0.0 (0.5)	0.1 (1.1)	<0.0001	0.1 (0.8)	0.4 (2.7)	<0.0001	<0.0001

^1^Values are medians (IQRs). Macronutrients are adjusted for total energy intake using either the nutrient residual (g) or nutrient density (percent energy) method

^2^*P *values compare differences in median intakes between races within region (i.e., black *vs*. white) using the Wilcoxon two-sample test.

^3^Global *P *values compare differences in median intakes across all three regions (i.e., Stroke Buckle *vs*. Stroke Belt *vs*. Other) and were calculated using the Kruskal-Wallis test

**Table 4:** Daily micronutrient intakes among 12,105 women participating in the Reasons for Geographic and Racial Differences in Stroke study, stratified by race and geographic region

**Micronutrients (intakes/d)^1^**	**Stroke Buckle**			**Stroke Belt**			**Other** **(non-stroke buckle or belt)**			***P *value between regions^3^**
	**Black** **(n = 971)**	**White** **(n = 1,905)**	***P*** **value^2^**	**Black** **(n = 1,600)**	**White** **(n = 2,603)**	***P*** **value^2^**	**Black** **(n = 2,237)**	**White** **(n = 2,789)**	***P*** **value^2^**	
**Vitamin A, IU**	7352 (6733)	7557 (6447)	0.34	6728 (6497)	7446 (6764)	0.0005	7154 (6517)	8225 (7186)	<0.0001	<0.0001
**Thiamin, mg**	1.1 (0.7)	1.1 (0.6)	0.47	1.1 (0.7)	1.1 (0.6)	0.12	1.1 (0.7)	1.1 (0.6)	0.04	0.03
**Riboflavin, mg**	1.2 (0.7)	1.3 (0.8)	<0.0001	1.2 (0.8)	1.4 (0.8)	<0.0001	1.2 (0.8)	1.4 (0.8)	<0.0001	0.01
**Niacin, mg**	14.4 (10.9)	15.7 (9.4)	<0.0001	14.8 (10.5)	16.3 (9.9)	<0.0001	14.5 (10)	16.4 (9.4)	<0.0001	0.17
**Folate, μg**	283 (200)	293 (178)	0.27	293.6 (203)	307 (181)	0.03	287 (191)	303 (170)	<0.0001	0.05
**Vitamin B-12, μg**	2.6 (2.2)	2.9 (2.2)	<0.0001	2.6 (2.3)	3.0 (2.3)	<0.0001	2.4 (2.1)	3.0 (2.2)	<0.0001	0.01
**α-tocopherol, μg**	7.4 (5.9)	8.2 (5.4)	<0.0001	7.8 (5.7)	8.6 (5.8)	<0.0001	7.9 (5.8)	8.6 (5.6)	<0.0001	0.0002
**β-carotene, μg**	2918 (2946)	2778 (2676)	0.18	2706 (2837)	2751 (2822)	0.98	2812 (2877)	2933 (3012)	0.21	0.0009
**Vitamin C, mg**	101 (96.3)	79.1 (73.0)	<0.0001	101 (96)	80.5 (76.0)	<0.0001	98.5 (92.7)	93.2 (78.1)	0.0002	<0.0001
**Vitamin D, IU**	83.0 (91.1)	105 (117)	<0.0001	90 (107)	113 (120)	<0.0001	86.8 (104)	111 (125)	<0.0001	0.07
**Calcium, mg**	460 (394)	572 (419)	<0.0001	506 (394)	606 (433)	<0.0001	485 (392)	643 (465)	<0.0001	<0.0001
**Magnesium, mg**	212 (137)	247 (140)	<0.0001	218 (138)	255 (147)	<0.0001	221 (143)	259 (141)	<0.0001	0.001
**Potassium, mg**	2093 (1362)	2361 (1276)	<0.0001	2148 (1346)	2464 (1341)	<0.0001	2169 (1356)	2570 (1310)	<0.0001	<0.0001
**Iron, mg**	9.7 (6.9)	10.5 (6.3)	0.0002	10.1 (7.0)	11.0 (6.8)	<0.0001	9.7 (6.5)	10.9 (6.3)	<0.0001	0.04
**Sodium, mg**	1779 (1352)	1889 (1112)	0.02	1854 (1356)	1947 (1186)	0.04	1806 (1274)	1980 (1081)	<0.0001	0.009
**Cholesterol, mg**	161 (159)	155 (113)	0.13	167 (150)	156 (124)	0.002	152 (142)	162 (124)	0.005	0.77

^1^Values are medians (IQRs). Micronutrients are adjusted for total energy intake using the nutrient residual method

^2^*P *values compare differences in median intakes between races within region (i.e., black *vs*. white) using the Wilcoxon two-sample test

^3^Global *P *values compare differences in median intakes across all three regions (i.e., Stroke Buckle *vs*. Stroke Belt *vs*. Other) and were calculated using the Kruskal-Wallis test

In linear regression analyses, significant interactions between race and region were detected for trans fat, calcium, and cholesterol (*p *< 0.05 for all) thus these analyses were stratified to obtain independent effects of race within region (Table 5). Blacks consumed 1.05% lower energy from saturated fat (95% CI: -0.95, -1.16), and intakes were also lower in the Buckle (β = -0.20%; 95% CI: -0.08, -0.32) and Belt (β = -0.35%; 95% CI: -0.24, -0.46) compared to the Other regions. Trans fat intake was significantly lower among blacks only in the Belt (β = -0.16%; 95% CI: -0.08, -0.25). Sodium, potassium, and magnesium intakes were all lower among black women compared to white women, and intakes were significantly lower among those living in the Belt and Buckle compared to those in the Other regions. Blacks consumed significantly less calcium than white women, with the largest effect among those living in the Other regions (β = -135.6 mg; 95% CI: -118.8, -152.4). Similarly, intakes of cholesterol were significantly lower for white women compared to black women only in the Other regions (β = -9.76 mg; 95% CI: -4.36, -15.15). We repeated multivariable analyses in a sub-sample of women with an income < $25,000 per year to further assess potential confounding by income. Results for women with an income < $25,000 per year were similar to those in the full sample (*data not shown*).

**Table 5:** Multivariable linear regression analyses showing associations between race, region, and nutrients among 12,105 women participating in the Reasons for Geographic and Racial Differences in Stroke study^1^

**Nutrient outcome**	**Effects of race and region on nutrient intakes**			
	**β**	**95% CI**	***P *value**	**Adjusted Means (SE)**
**Fiber (g)**				
Black	-0.37	(-0.12, -0.61)	0.003	15.1 (0.09) vs. 15.5 (0.07) [Black vs. white]
Stroke Buckle	-0.61	(-0.33, -0.88)	<0.0001	15.0 (0.1) vs. 15.6 (0.08) [Stroke Buckle vs. Other]
Stroke Belt	-0.42	(-0.17, -0.66)	0.0009	15.1 (0.08) vs. 15.6 (0.08) [Stroke Belt vs. Other]
**Saturated fat** **(% energy)**				
Black	-1.05	(-0.95, -1.16)	<0.0001	10.0 (0.04) vs. 11.0 (0.03) [Black vs. white]
Stroke Buckle	-0.20	(-0.08, -0.32)	<0.0009	10.5 (0.05) vs. 10.7 (0.04) [Stroke Buckle vs. Other]
Stroke Belt	-0.35	(-0.24, -0.46)	<0.0001	10.3 (0.04) vs. 10.7 (0.04) [Stroke Belt vs. Other]
**Trans fat** **(% energy)^2^**				
Stroke Buckle: Black	-0.06	(-0.16, 0.03)	0.20	2.92 (0.04) vs. 2.98 (0.03) [Black vs. white]
Stroke Belt: Black	-0.16	(-0.08, -0.25)	0.0002	2.80 (0.02) vs. 3.12 (0.03) [Black vs. white]
Other: Black	-0.007	(-0.08, 0.06)	0.84	2.80 (0.02) vs. 2.80 (0.03) [Black vs. white]
**Sodium (mg)**				
Black	-85.23	(-67.6, -102.9)	<0.0009	2042 (6.7) vs. 2122 (5.3) [Black vs. white]
Stroke Buckle	-36.43	(-16.52, -56.33)	0.003	2067 (8.1) vs. 2103 (6.1) [Stroke Buckle vs. Other]
Stroke Belt	-36.84	(-18.95, -54.74)	<0.0001	2067 (6.7) vs. 2103 (6.1) [Stroke Belt vs. Other]
**Potassium (g)**				
Black	-228.3	(-202.7, -253.9)	<0.0001	2375 (9.7) vs. 2593 (7.6) [Black vs. white]
Stroke Buckle	-98.50	(-69.7, -127.3)	<0.0001	2438 (11.7) vs. 2536 (8.9) [Stroke Buckle vs. Other]
Stroke Belt	-85.30	(-59.4, -111.2)	<0.0001	2451 (9.7) vs. 2536 (8.9) [Stroke Belt vs. Other]
**Magnesium (mg)**				
Black	-22.67	(-19.92, -25.43)	<0.0001	246 (1.0) vs. 268 (0.82) [Black vs. white]
Stroke Buckle	-7.11	(-4.01, -10.21)	<0.0001	253 (1.3) vs. 261 (0.95) [Stroke Buckle vs. Other]
Stroke Belt	-5.49	(-2.70, -8.28)	0.0001	255 (1.0) vs. 261 (0.95) [Stroke Belt vs. Other]
**Calcium (mg)^2^**				
Stroke Buckle: Black	-96.82	(-74.97, -118.7)	<0.0001	540 (8.7) vs. 637 (5.9) [Black vs. white]
Stroke Belt: Black	-97.83	(-78.90, -116.8)	<0.0001	579 (7.2) vs. 675 (5.5) [Black vs. white]
Other: Black	-135.56	(-118.8, -152.4)	<0.0001	565 (6.1) vs. 701 (5.3) [Black vs. white]
**Cholesterol (mg)^2^**				
Stroke Buckle: Black	-1.22	(-8.61, 6.17)	0.75	183 (2.9) vs. 185 (2.0) [Black vs. white]
Stroke Belt: Black	-1.81	(-7.84, 4.22)	0.56	186 (2.3) vs. 188 (1.7) [Black vs. white]
Other: Black	-9.76	(-4.36, -15.15)	0.0004	180 (2.0) vs. 190 (1.7) [Black vs. white]

^1^For unstratified analyses, the reference group for race is white and the reference group for region is "other." All models are adjusted for the following covariates: age, total energy, BMI, multivitamin use, menopausal status, hormone therapy use, income, education, marital status, smoking status, alcohol use, physical activity, television viewing, and diagnoses of disease (hypertension, hypercholesterolemia, and diabetes).

^2^A significant interaction between race and region was detected for trans fat (*p *= 0.0014), calcium (*p *< 0.0001), and cholesterol (*p *= 0.006). These analyses were stratified by region and beta coefficients represent effects for blacks (referent: white)

## Discussion

Black Americans and those who reside in the Stroke Belt and Buckle have a greater risk of stroke [10,19], and diet is related to the risk of several chronic diseases in these population sub-groups [1,2]. Our study is the first to examine dietary intakes among women in the REGARDS population with the hope that this work will lead to generation of hypotheses about how diet might be related to health disparities.

A major goal of this study was to disentangle the effects of race and region among black and white women living in the Stroke Belt, Stroke Buckle, and Other regions in the United States. Our results showed lower intakes of many key nutrients among blacks compared to non-Hispanic whites, as well as lower intakes in the Belt and Buckle. For intakes of trans fat, calcium, and cholesterol, the effect of region on nutrient intake was modified by race, showing significant differences comparing black to white women only in certain regions. Therefore, race and region have both independent and synergistic effects on diet and their effects may be mediated by diverse cultural influences as well as environmental factors influencing food availability, accessibility, and eating behaviors. For example, intakes of cholesterol were lower only among black women in the Other regions, but not in the Buckle and Belt. This implies that perhaps intakes of cholesterol in Southern diets are similar for women due to regional effects on diet, regardless of race, whereas significant racial differences in cholesterol intake between black and white women are seen only in Other regions, where a Southern dietary pattern is likely not as prominent. The United States is diverse and has many cultures with unique eating preferences, thus attention to both region and race and their correlates is needed when trying to understand what people are eating and why. This information is critical in creating effective dietary guidance for population sub-groups.

While lower intakes of most nutrients were observed in the Belt and Buckle, blacks consistently consumed lower amounts of nutrients compared to whites, independent of region, as seen in our companion paper among men [17]. Black women consumed less fiber, potassium, sodium, and saturated fat and more cholesterol in one study [3] while another showed lower calcium and magnesium intakes [7]; region was not considered in these studies. Hajjar and Kotchen [5] considered the role of region, but not race, on nutrient intakes, finding higher cholesterol and lower calcium, potassium, and magnesium intakes among Southern adults compared to other regions. In our study, fiber intakes were much lower than the recommended 14 g/1000 kcal [20] across all race/region groups, with no clinically meaningful differences across strata. Although FFQs are not able to provide accurate measures of absolute nutrient intakes, our results of low fiber intakes are consistent with Champagne et al. [8], who studied a representative sample of adults in the United States and the Mississippi Delta. Specifically, blacks within the Delta consumed significantly less fiber, potassium, magnesium, and calcium than whites. In both racial groups, those living in the Delta consumed less of these same nutrients when compared to their respective racial groups in the general United States population.

We observed significant differences in fat intakes across race and region, but the body of literature in this area is limited and conflicting. Intake of saturated fat in our study was 1.05% lower among blacks and was also lower in the Buckle and Belt compared to those in the Other regions. Diaz et al. [3] similarly found that blacks consumed 2.0% less saturated fat than white overweight adults, while another study found blacks consumed more saturated fat than whites [21]. Southerners participating in NHANES III consumed ≈ 1.3 kJ/d more total fat than those elsewhere [5], although the study did not consider race in its analysis. No differences in saturated fat intake were seen comparing women in the Delta to a national sample of adults in the United States for either racial group [8]. Trans fat intakes were lower among blacks in our study, but only in the Belt. Although the estimate for this effect is small - 0.16% lower energy for trans fat for blacks compared to whites in the Stroke Belt - it is nonetheless meaningful given the American Heart Association's recommendation to limit trans fat to <1% of daily energy [22]. It is unclear why this effect was observed only for the Belt and not the Buckle, given the effects for many other nutrients were similar between these two regions. We know of no other study that has examined trans fat intakes across race and region subgroups, and additional research is needed.

It was interesting that the majority of our analyses where interactions were not present (saturated fat, sodium, potassium, magnesium) showed stronger effects for race compared to region. While significant regional effects also were observed, estimates between the Buckle and Belt are similar for most analyses but are often half the magnitude of the effect of race. In another study in the REGARDS cohort, Cushman et al. [19] also showed smaller regional differences in stroke risk factors such as blood pressure and hypertension compared to the effect of race. It is not clear why stronger effects for race/ethnicity were observed compared to region in this study and ours, but results perhaps suggest that innate and/or learned preferences for dietary intakes and food behaviors among blacks transcend the effect of geographic region.

We elected to begin our examination of the dietary data in this population with nutrient intakes before expanding to the food, food group, and dietary patterns levels. However, individuals may choose from a variety of different foods and reach the same nutrient intake. For example, Mississippi Delta blacks had very similar intakes of total fat compared to white women, but blacks consumed more fat from fried chicken and sausage and whites consumed more fat from salad dressings and cheese [23]. Similarly, individuals consuming a diet high in white bread and refined grains had a similar percentage of energy intake from carbohydrates compared to those consuming a healthy, plant-based pattern [24]. We anticipate that our future projects examining foods and diet patterns will provide meaningful insights into race and regional differences in diet and associations with disease.

Strengths of our study include its sample size, large number of blacks, and regional data that includes the Belt and Buckle. We also adjusted our analyses for many important covariates, which were important in isolating unbiased effects of race and region on dietary intakes although residual confounding by other factors is always a possibility in epidemiologic studies. We performed an additional analysis among women with incomes < $25,000 and results were similar to those in the full sample, thus it is unlikely that the observed differences are a result of residual confounding by socioeconomic status. Whereas several studies have looked at race, region, or sex in separate analyses, we know of no other studies that specifically examined together the effects of race and region on dietary intakes in a large population of women using rigorous, multivariable analyses. Our study also has several limitations. This study is a cross-sectional analysis, although the potential for reverse causality is unlikely for our main exposures and outcomes (i.e., nutrient intake can not predict race or region). It is possible that disease status may have impacted our results, although we adjusted for several baseline diseases to reduce this potential bias. Selection bias may have resulted from using commercially available lists for recruitment, as not all individuals have listed telephone numbers and/or a mailing address. We are limited by the use of the Block 98 FFQ, which is now relatively old; however, this was among the main tools used to collect dietary data on racially diverse populations at the time this study was conceived (c. 2000) and it specifically included foods contributing to diets among blacks living in the United States (personal communication, Torin Block, NutritionQuest™, Berkeley, CA). While this FFQ has been validated in a different population of women [15], it has not yet been validated in the REGARDS population, as aforementioned. FFQs were not designed to measure absolute intakes, and estimates for some of our micronutrients were lower than expected. Using diet records in a small metabolic study, Lovejoy et al. [7] reported similar means to ours for calcium, but other nutrients were generally higher than those we reported, as seen elsewhere [8,25-27]. Under-reporting is a common error for self-reported dietary instruments, especially the FFQ, and both random and systematic under-reporting may have had an even greater impact in this population, many of whom were older and of lower SES [28] and were overweight or obese [29]. It is also important to remember that while our large sample allowed us to detect (highly) statistically significant differences in nutrient intakes, many of these differences are quite small. Relying upon statistical significance can be misleading and some of our findings are likely of limited clinical importance. Nonetheless, along with our companion study in men [17], ours are the first studies to demonstrate that race and region interact together in influencing dietary intakes, underscoring the complexity of eating behavior. Finally, while our analysis showed interesting findings of effect modification (race*region) on nutrient intakes, and our findings appear reasonable, interaction effects can be difficult to replicate [16]; additional research is needed to confirm these results.

## Conclusions

Our study showed that race and region were significantly associated with nutrient intakes in a large study of black and non-Hispanic white women in the United States and that, in some cases, the effect of region was modified by race. Race and region thus interact to impact dietary intakes, and their effects may be mediated by such factors as the broader food environment and food availability as well as food customs and culture. Race, region, and their correlates should therefore be considered together when examining diet and disease associations and planning dietary advice for population sub-groups.

## Abbreviations

CATI: Computer-assisted telephone interview; FFQ: food frequency questionnaire; NHANES: National Health and Nutrition Examination Survey; REGARDS: Reasons for Geographic and Racial Differences in Stroke.

## Competing interests

The authors declare that they have no competing interests.

## Authors' contributions

PKN was responsible for conception and design of this study; PKN, SJ, SEN, and RG all contributed to statistical programming; SJ, SEN, and PKN analyzed data; RG, SEN, and PKN wrote the paper; JMS, SJ, and JA provided feedback on all analyses and made critical comments on the manuscript. All authors participated in the preparation of the final manuscript and critically reviewed and approved the manuscript for publication. 
